# Supplementary material for: EEG/fNIRS Based Workload Classification Using Functional Brain Connectivity and Machine Learning
Source: Sensors (Basel). 2022 Oct 8;22(19):7623. doi: 10.3390/s22197623 (PMC9571712; doi:10.3390/s22197623)
Supplement: Supplementary file 1 [file sensors-22-07623-s001.zip › sensors-1919026-supplementary.pdf]

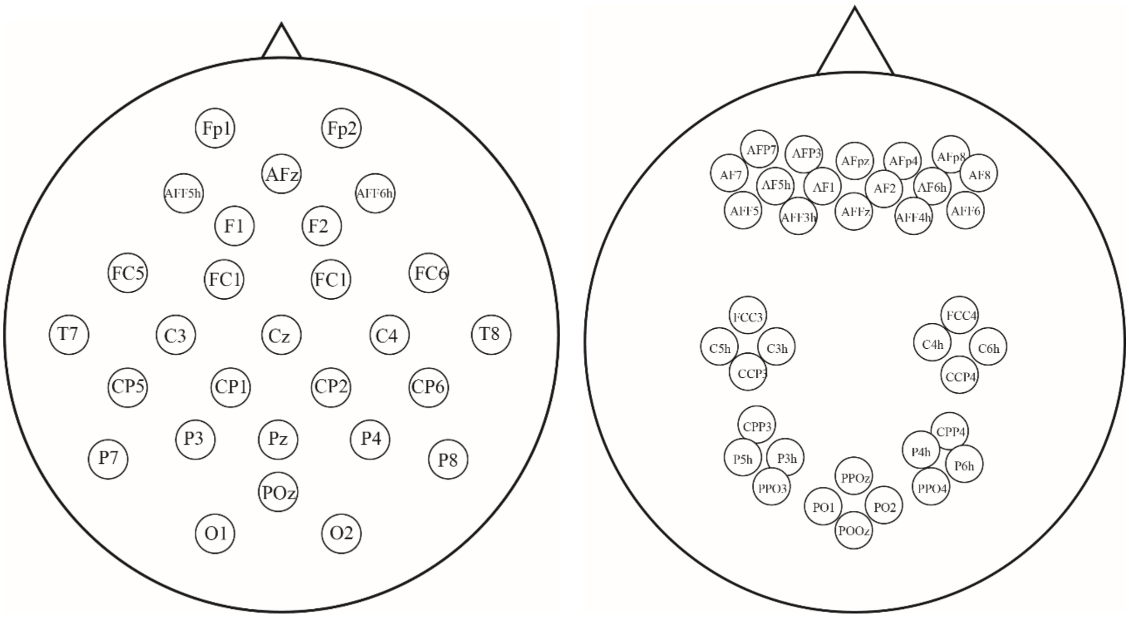


*Figure S1. Channels and locations for the EEG (Left) and fNIRS (Right) recordings*

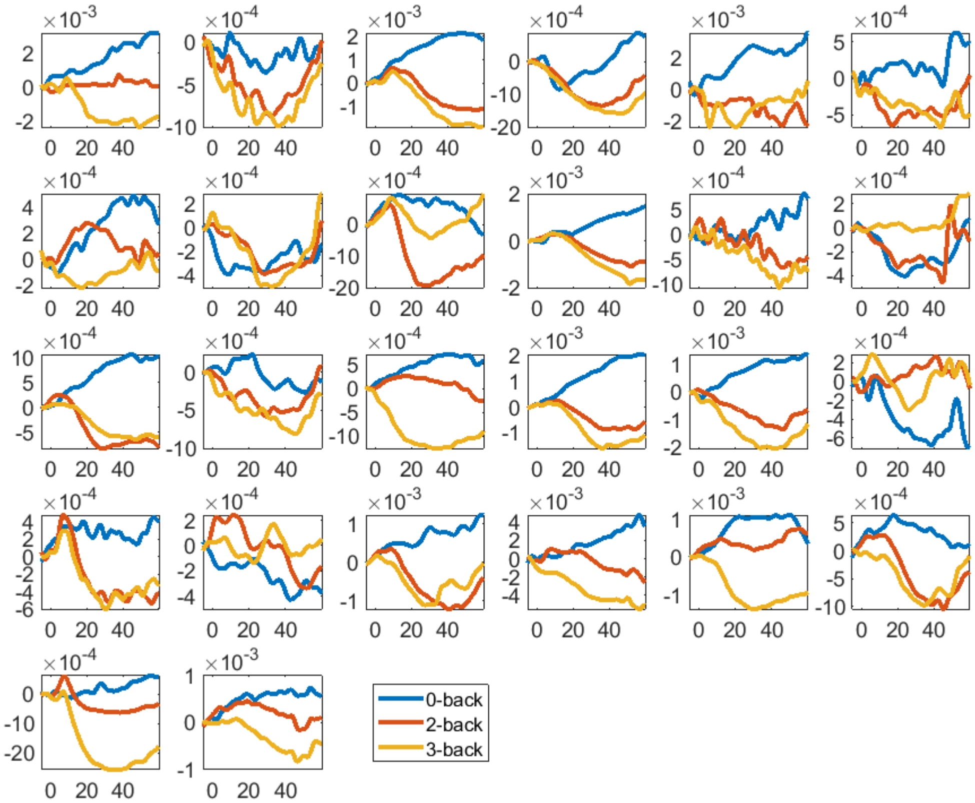


*Figure S2. fNIRS average HbR value of each of 26 participants in three levels of workload*

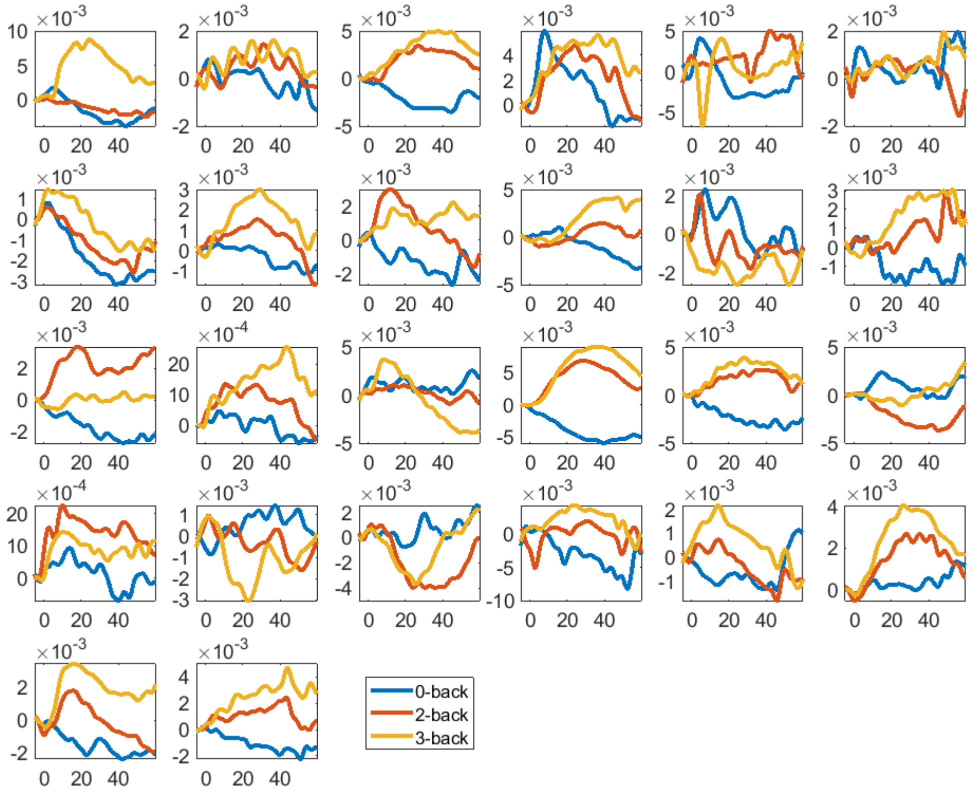


*Figure S3. fNIRS average HbO value of each of 26 participants in three levels of workload*

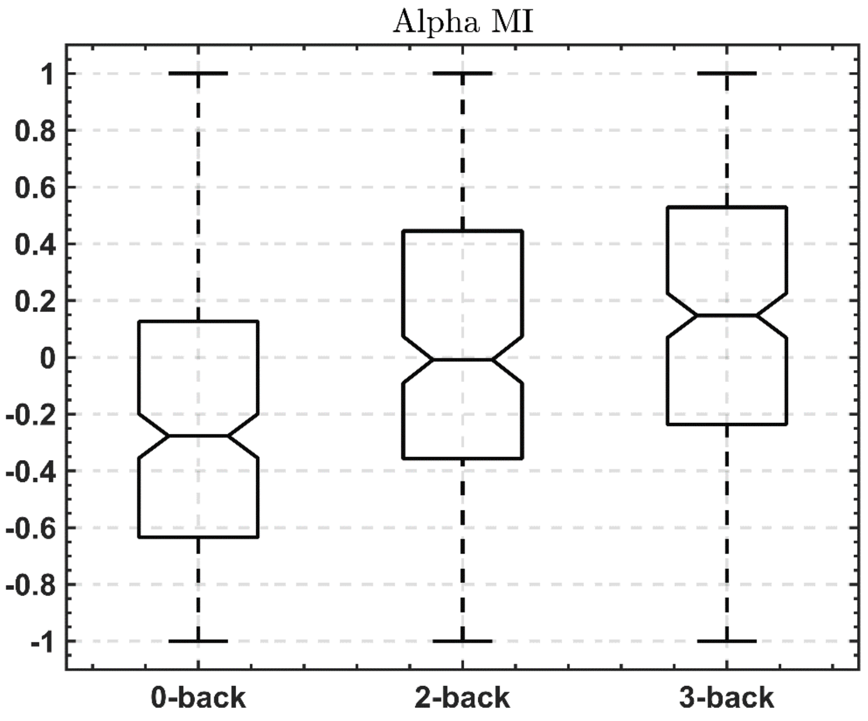


*Figure S4. A sample of significant test to represent the difference among three-level workload with the purpose of selecting limited numbers of features. (p-value<0.0001)*
